# New Insights into Heavy Metal Sequestration Through Metal‐Phenolic Network‐Confined Nano‐HFO: Overlooking Iron Utilization and Modulating Electron Density

**DOI:** 10.1002/advs.202417798

**Published:** 2025-03-28

**Authors:** Manyu Zhang, Xiaolin Du, Zhanqi Liu, Yujia Yang, Shuo Wang, Ningyi Chen, Yulin Wang, Yaran Song, Keju Sun, Qingrui Zhang

**Affiliations:** ^1^ State Key Laboratory of Metastable Materials Science and Technology Hebei Key Laboratory of Heavy Metal Deep‐remediation in Water and Resource Reuse Yanshan University Qinhuangdao 066004 China; ^2^ Hong Qi Sheng Precision Electronics (Qinhuangdao) Co., LTD Qinhuangdao 066004 China; ^3^ College of Environment Zhejiang University of Technology Hangzhou Zhejiang 310014 China; ^4^ Hebei Province Engineering Research Center for Harmless Synergistic Treatment and Recycling of Municipal Solid Waste Yanshan University Qinhuangdao 066004 China

**Keywords:** adsorption, DFT calculation, heavy metal, metal‐phenolic network

## Abstract

Reducing toxic metal concentrations to trace levels remains a critical challenge in water remediation, largely due to the underutilization of hydrous ferric oxide (HFO), particularly within its inner layers. Herein, we present a novel strategy to enhance HFO utilization by in situ confinement of nano‐HFO within polystyrene beads using a tannic acid‐zirconium (TA‐Zr) metalphenolic network, forming PS‐Fe@TA‐Zr. The TAZr network generates a highly negative microenvironment with tunable electron density at oxygen sites, facilitating Pb(II) enrichment and activating inner‐layer Fe sites. Depth‐profiling reveals a significant increase in the Pb/Fe ratio from 7.6% at the surface to 18.8% at 10 nm depth, highlighting the contribution of previously inaccessible active sites. The TAZr confinement also modulates electron density at Fe and O sites, enabling stronger hybridization with Pb 4f orbitals and enhancing Pb(II)HFO interactions. Compared with PS‐Fe, PS‐Fe@TA‐Zr exhibits over 8‐fold higher selectivity (Kd = 15,278 mL g^−1^), 5‐fold faster kinetics, and can treat up to 1,680 L kg^−1^ with effective regeneration across six cycles in actual industrial wastewater. This work provides new insights into metalphenolic network‐assisted design of nanocomposites for highly efficient iron utilization in heavy metal removal.

## Graphical abstract

Iron‐based nanocomposites are widely used for trace heavy metal removal, but the low utilization of HFO nanoparticles remains a challenge. Here, a metal‐phenolic network (MPN) strategy is proposed to confine nano‐HFO within polystyrene beads, creating a negative electrostatic field and optimizing electron density at oxygen sites. This approach enhances iron utilization, achieving over 8‐fold higher selectivity (K_d_ = 15,278 mL g^−1^), 5‐fold faster kinetics, and treating 1,680 L kg^−1^, with effective regeneration across 6 cycles in industrial wastewater.

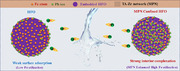

## 1 Introduction

Heavy metal contamination from industrial activities such as mining, smelting, and chemical production has become a critical environmental issue.^[^ ^1^ ^]^ These metals, even at trace concentrations, easily migrate through aqueous environments, entering the food chain and bioaccumulating organisms, which poses significant risks to human health.^[^ ^2^ ^]^ Technologies for capturing heavy metal ions include adsorption,^[^ ^3^ ^]^ chemical precipitation,^[^ ^4^ ^]^ membrane separation,^[^ ^5^ ^]^ and electrochemical methods.^[^ ^6^ ^]^ Chemical precipitation is effective for high concentrations but less so for trace levels,^[^ ^7^ ^]^ while membrane separation struggles in high‐salinity environments.^[^ ^8^ ^]^ Conversely, adsorption using nanosized adsorbents demonstrates considerable potential for reducing metal concentrations to trace levels.^[^ ^9^ ^]^

Metal oxide nanoparticles (MONPs) like Fe(III),^[^ ^10^ ^]^ Zr(IV),^[^ ^11^ ^]^ and Mn(IV)^[^ ^12^ ^]^ are highly effective in pollutant removal due to their size‐dependent activity and robust inner‐sphere complexation.^[^ ^13^ ^]^ However, the application of HFO nanoparticles in actual wastewater treatment faces challenges due to their high dynamic resistance and difficulties in separation and recycling.^[^ ^14^ ^]^

An engineering alternative involves developing nanocomposites that encapsulate metallic oxide nanoparticles in porous polystyrene resins, providing a scalable solution for heavy metal contamination in practical applications.^[^ ^11^ ^]^ However, in engineering, there is often an overemphasis on application performance, resulting in little attention to the effective utilization of nanoparticles. In fact, incorporating nano‐HFO within matrices often obscures Fe utilization due to spatial restrictions and interaction dynamics. For example, polymer nanocomposites incorporating HFOs (3% to 17% Fe by mass) showed varying arsenate sorption behaviors; higher Fe loadings (≈17%) significantly reduced sorption capacity, while nanocomposites with lower HFO content (<3%) exhibited stronger sorption affinity at trace arsenate concentrations (<1 mg L^−1^).^[^ ^15^ ^]^ These findings collectively highlight the often‐overlooked significance of understanding nanoparticle utilization.

Metal‐Polyphenol Networks (MPNs) are eco‐friendly coating materials synthesized rapidly through the coordination of metal ions with polyphenol ligands.^[^ ^16^, ^17^, ^18^ ^]^ A prominent example is the complexation of tannic acid (TA) with metals such as Fe^[^ ^19^ ^]^ or Zr.^[^ ^20^ ^]^ TA, a green and safe food‐grade compound, derived from organic agricultural waste (e.g., fruit, vegetable peels, leaves, seeds), enables efficient resource utilization.^[^ ^21^ ^]^ TA ligands impart strong electronegativity due to the ionization of catechol and gallic acyl groups, which is highly pH‐dependent. At pH 2, the ionization degree is 7.58 × 10^−4^, ≈100 times lower than at pH 4 (7.05 × 10^−2^). At pH 5, it increases to 0.43, and at pH 7, TA approaches complete ionization.^[^ ^22^ ^]^ Moreover, the strong electronegativity of TA is supported by zeta potential measurements. Frank Caruso et al. reported a decrease in the zeta potential of polystyrene (PS) from −27 ± 3 to −64 ± 7 mV after TA‐based MPN coatings, a trend observed across various substrates such as glass, Au, SiO_2_, CaCO_3_, and even microbial Escherichia coli.^[^ ^20a^ ^]^ This enhanced electronegativity makes MPNs effective coatings for heavy metal ion enrichment, significantly improving the diffusion efficiency and utilization of HFO nanoparticles in nanocomposites. Furthermore, the cross‐linked network structure of MPN forms nanoconfined HFO domains, reducing the activation energy for heavy metal interactions. MPNs facilitate electron transfer via oxygen bridges, lowering electron cloud density and exposing more active sites under MPN confinement, This enhances the alignment of Fe 3d and Pb 6p orbitals, promoting the formation of strong Fe─O─Pb bonds, thereby enabling efficient Pb(II) ion removal and maximizing the utilization of nano‐HFO.

In this study, TA‐Zr was developed as a representative metal‐phenolic network strategy to significantly enhance iron utilization efficiency through the in situ growth of nano‐HFO within polystyrene beads. The TA‐Zr networks form a confined nano‐HFO space with a highly negative environment, inducing substantial alterations in the electron distribution and coordination structure of the nano‐HFO. This charged modification facilitates the preferential enrichment and accelerated diffusion of heavy metal ions, markedly improving nano‐HFO utilization and purification efficiency. Moreover, the nanoconfinement provided by the MPN structure acts as an electron transfer conduit, reducing the electron cloud density and further reinforcing the coordination interactions with heavy metals.

Pb(II) ions‐chosen due to their high toxicity and stringent regulatory discharge limits‐were employed to assess decontamination performances, using PS─Fe as a comparative baseline. This study emphasizes the differential enhancement of nano‐HFO utilization and the intensification of heavy metal coordination, driven by the charged MPN confinement. These effects were validated through practical engineering applications. To further unravel the underlying mechanisms, XPS with ion sputtering, in conjunction with DFT calculations, was employed to probe the depth‐dependent utilization efficiency and coordination dynamics within the nanoparticles. This comprehensive analysis is intended to provide critical insights that will inform the future design and application of these nanocomposite materials.

## 2 Results and Discussion

### 2.1 Characterization

**Figure 1:**
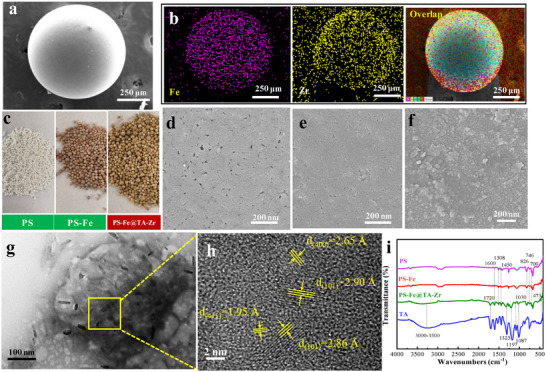
a,b) SEM image and EDX elemental mapping of PS─Fe@TA‐Zr. c) The optical photos of PS─Fe@TA‐Zr. d–f) SEM images of PS, PS─Fe and PS─Fe@TA‐Zr. g) TEM images of PS─Fe@TA‐Zr. h) High‐resolution TEM image of PS─Fe@TA‐Zr. i) FT‐IR of TA, PS, PS─Fe and PS─Fe@TA‐Zr.

The typical morphology of the produced PS─Fe@TA‐Zr was first examined by scanning electron microscopy (SEM; **Figure** **1**a,b,d–f) and transmission electron microscopy (TEM; Figure 1g,h). The bare PS framework is spherical, with a diameter of ≈0.7 mm (Figure 1a), and has a porous polystyrene matrix (Figure 1d). In contrast, the PS‐Fe composite exhibited a denser surface coating with noticeable pore blocking, likely due to the formation of HFO (Figure 1e). This phenomenon indicates a potential weakening of sorption diffusion during heavy metal removal, thereby reducing the efficiency of nano‐HFO utilization. Importantly, the assembly of the TA‐Zr network on the PS─Fe surface significantly increases roughness, revealing a distinct crystalline nanorod morphology with a size of ≈10–20 nm (Figure 1f). To further verify the distribution of HFO and TA‐Zr network, SEM‐EDS mapping analysis was conducted, showing a uniform dispersion of C, O, Cl, Fe, and Zr elements throughout the PS matrix (Figure 1b). Also, the color of PS matrix changed from white to reddish brown, after the in situ formation of HFO (Figure 1c). Assembling the TA‐Zr network, it further changed to yellow (Figure 1c). The TEM image further confirms the existence of a large number of rod‐shaped nanoparticles, with a length of ≈30 nm and a width of ≈10 nm (Figure 1g) and the nanoparticle exhibits a crystal structure with lattice spacing of 1.95, 2.65, 2.8, and 2.90 Å, respectively by HR‐TEM investigation, corresponding to the (411), (400), and (101) crystal planes of FeO(OH) (JCPDS No. 75–1594), thereby confirming the formation of HFO nanoparticles (Figure 1h).

FTIR spectral analysis was conducted to verify the surface components of the PS, PS─Fe, and PS─Fe@TA‐Zr nanocomposite (Figure 1i). The peaks at 1600, 1508, and 1450 cm^−1^ are associated with the C═C stretching vibration in the benzene ring of the PS,^[^ ^23^ ^]^ while the peak at 826, 746, and 700 cm^−1^ corresponds to the absorption region of the C─H out‐of‐plane bending vibration in the benzene ring.^[^ ^24^ ^]^ Notably, a new characteristic peak at 473 cm^−1^ belongs to the Fe─O bond,^[^ ^25^ ^]^ indicating that HFO nanoparticles have been successfully loaded onto the PS─Fe samples. After the modification of the TA‐Zr network, the characteristic peaks of TA were prominently observed. The wide peak at 3000 ≈3500 cm^−1^ can be attributed to the stretching vibration of a large amount of O‐H within TA.^[^ ^26^ ^]^ The observed peaks at 1720 and 1030 cm^−1^ are related to the C═O stretching vibration and the antisymmetric stretching of C─O─C in the pyran ring, respectively. Additionally, the peaks at 1325, 1197, and 1087 cm^−1^ correlate with the stretching vibration of C─O of TA.^[^ ^27^ ^]^ Moreover, the XRD pattern reveals a low crystallinity of HFO in both PS─Fe and PS─Fe@TA‐Zr nanocomposites (Figure S1a, Supporting Information). This suggests that heavy metals facilitate entry into the structure during the adsorption process.

XPS further verified and tracked the HFO loading and the subsequent TA‐Zr enrichment process on the surface of nanocomposites (Figure S1b–d, Supporting Information). The PS matrix initially showed peaks for only C and Cl. Following the in situ synthesis of nano‐HFO, characteristic peaks for O 1s and Fe 2p emerged (Figure S1b, Supporting Information). Specifically, peaks at 723.8 and 710.3 eV correspond to Fe^III^ 2p_1/2_ and Fe^III^ 2p_3/2_,^[^ ^28^ ^]^ respectively (Figure S1c, Supporting Information). The peaks at 725.9 and 712.5 eV are associated with the Fe─O (29%). After the modification of the TA‐Zr network, the proportion of Fe─O increases significantly (from 29% to 35%), suggesting the interaction of HFO and TA‐Zr network to form a new Fe─O─Zr bond. Additionally, distinct Zr 3d_3/2_ and Zr 3d_5/2_ peaks appeared at 184.8 and 182.5 eV (Figure S1d, Supporting Information), indicating the presence of the TA‐Zr network in the PS─Fe@TA‐Zr nanocomposite.^[^ ^11^ ^]^ Notably, a pair of new peaks appeared at 185.5 and 183.4 eV, which can be attributed to the interaction of the TA‐Zr network and HFO to form a new Zr─O─Fe bond, which is consistent with the results observed in the Fe2p orbit. Furthermore, the significant enhancement of the O 1s peak in the XPS spectrum of PS─Fe@TA‐Zr, due to the numerous oxygen‐containing functional groups on the TA molecules, further confirms the successful loading of the TA‐Zr coating (Figure S1b, Supporting Information).

The pore size distributions, surface area, and pore volume of PS, PS─Fe, and PS‐Fe@TA‐Zr were summarized in Figure S2 and Table S1 (Supporting Information). PS matrix possesses a mesoporous structure with an average pore size of 30.64 nm. Whereas HFO and TA‐Zr coating onto PS significantly decreases the pore diameter of PS, the average pore size is decreased to 22.15 nm, which is possibly ascribed to the HFO occupation inside PS (Figure S2f, Supporting Information). In addition, the TA‐Zr immobilization also results in a distinct enhancement in BET surface area (from 51.20 to 60.58 m^2^ g^−1^) and pore volume (from 0.295 to 0.335 cm^3^ g^−1^), which might be due to the cross‐linked TA‐Zr network structure (Figure S2 and Table S1, Supporting Information).

### 2.2 Charged MPN Driven Solution Chemistry for Pb(II) Removal

**Figure 2:**
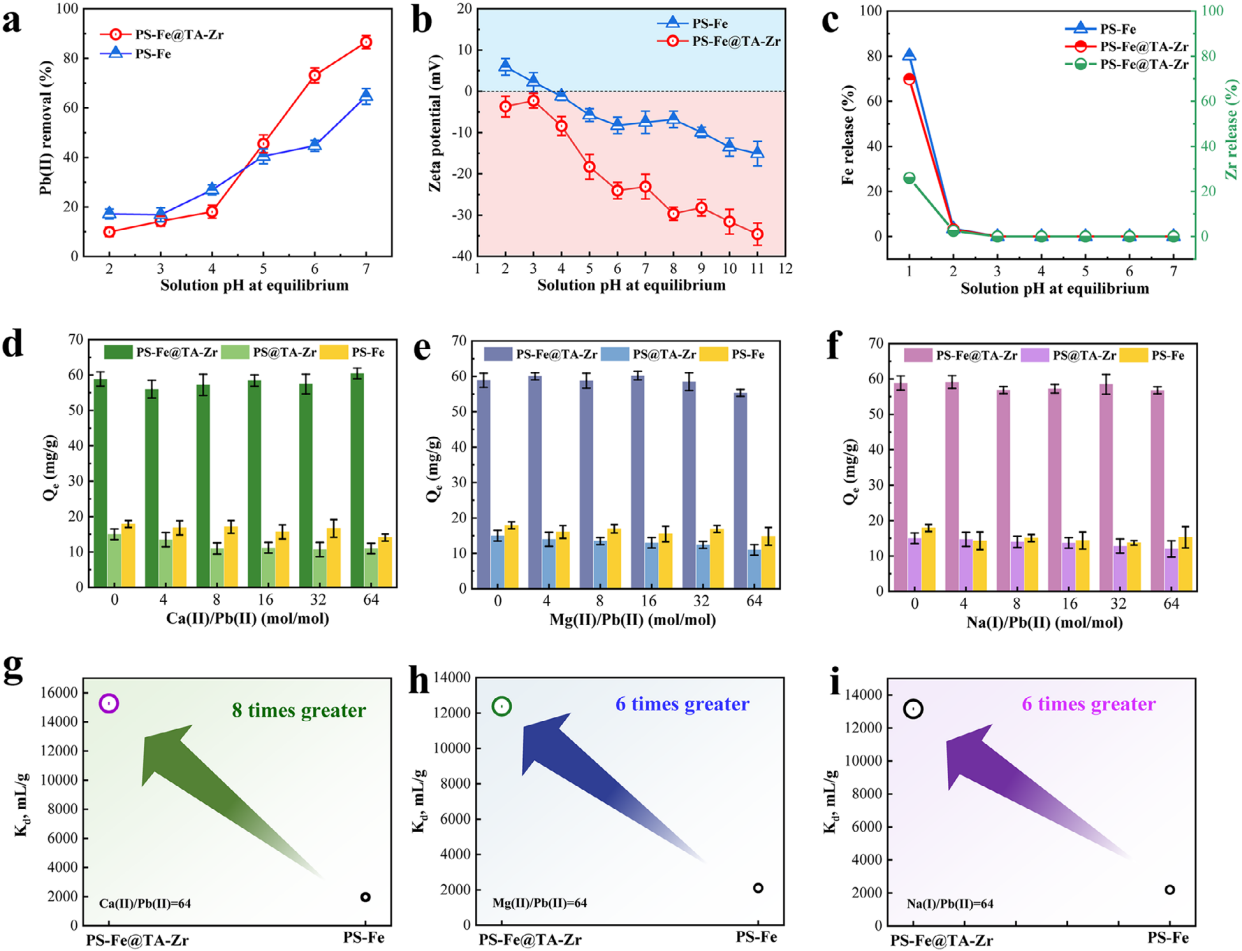
a) Effect of solution pH on removal of Pb(II) by PS─Fe and PS─Fe@TA‐Zr nanocomposite. Conditions: 0.1 g L^−1^ absorbents, initial Pb(II) = 10 mg/L, 50 mL solution, 298 K for 12 h reaction. b) The zeta potential charges at different solution pHs. c) Fe release of PS─Fe and PS─Fe@TA‐Zr, and Zr release of PS─Fe@TA‐Zr, 298 K, 0.1 g L^−1^ PS─Fe@TA‐Zr. d–f) Effect of competitive comparisons. d) Ca(II); e) Mg(II); f) Na(I). Conditions: 0.1 g L^−1^ adsorbents, initial Pb(II) = 10 mg L^−1^, 50 mL solution, pH = 5.5–6.0, 298 K. g–i) the K_d_ values comparisons of PS─Fe and PS─Fe@TA‐Zr nanocomposite at the condition of M(II)/Pb(II) = 64 (M representing Ca, Mg, or Na).

Industrial wastewaters from electroplating, metallurgy, and battery manufacturing often experience pH fluctuations due to the diverse operational conditions, which can alter the surface charge and adsorption capacity of adsorbents, thus affecting their performance in heavy metal removal. To address this, we performed a detailed investigation of the pH‐dependent adsorption performance of PS─Fe and PS─Fe@TA‐Zr nanocomposites across a pH range of 1.0 to 7.0. As shown in **Figure** **2**a, the adsorption capacity for Pb(II) is significantly affected by the solution pH, with higher pH levels greatly improving the adsorption process. Notably, at pH values above 6.0, PS─Fe@TA‐Zr demonstrates a superior adsorption efficiency for Pb(II), reaching up to 85%, compared to ≈60% for PS─Fe.

These distinct performances are primarily due to the pronounced electronegativity of the charged TA‐Zr confinement. Figure 2b shows that the TA‐Zr network significantly increases the negative surface charge from −15 ± 3 to −35 ± 5 mV, greatly enhancing the diffusion and enrichment of Pb(II) ions. This improvement markedly enhances the overall purification capability for heavy metals. Additionally, we observed negligible sorption capability under acidic conditions (pH 2.0–3.0), likely due to strong competition from H^+^ ions and the decomplexation of the TA‐Zr network from 6‐coordinate to 2‐coordinate structures.^[^ ^3b^ ^]^ Furthermore, the reduced electronegativity (approaching zero) also inhibited Pb(II) adsorption in acidic environments.

Furthermore, we assessed the stability of the adsorbents across different pH solutions (Figure 2c). Both PS─Fe and PS─Fe@TA‐Zr nanocomposites demonstrated effective stability with negligible Fe release at pH 2.0–7.0. However, substantial HFO dissolution was observed under strongly acidic conditions (pH = 1.0), indicating limited applicability in highly acidic wastewater. Additionally, the decomplexation of the TA‐Zr network was evaluated through Zr release performance. The negligible Zr release at pH 2.0–7.0 confirms the excellent stability and safety of the PS─Fe@TA‐Zr nanocomposite for purifying Pb(II) in wastewater (Figure 2c).

### 2.3 MPN Improved Pb(II) Selectivity and Fe Utilization Mechanism

#### 2.3.1 MPN Driven Selectivity Performances

Salinity is a common characteristic of industrial wastewater, where coexisting ions such as Ca(II), Mg(II), and Na(I) can compete with the adsorption of target pollutants like Pb(II).^[^ ^28^ ^]^ Therefore, achieving high selectivity for adsorbents is essential for effectively removing Pb(II) in treatment scenarios. In a study, PS─Fe, PS@TA‐Zr, and PS‐Fe@TA‐Zr nanocomposites demonstrated excellent selectivity for Pb(II), with minimal loss of adsorption capacity even in the presence of these competitive ions. Notably, the M(II): Pb(II) ratio (M representing Ca, Mg, or Na) reached as high as 64:1, highlighting the strong preference for Pb(II) over other coexisting ions (Figure 2d–f). This behavior can be ascribed to specific adsorption toward Pb(II) for cross‐linked confined HFO structures on the PS─Fe@TA‐Zr. The oxygen site at PS─Fe@TA‐Zr shows obvious specific adsorption of Pb(II), which can be inferred from the electron energy and the Pb─O binding energy calculated by DFT. Compared with PS─Fe, PS─Fe@TA‐Zr exhibits the lowest electron energy (−49431.3 eV) and the highest Pb─O binding energy (≈2498.2 kJ mol^−1^) for the adsorption of Pb(II), which will reveal the transformation of HFO structure and electron configuration in the limited domain of TA‐Zr networks, and provides evidence for its high selectivity for Pb(II).

Indeed, under identical conditions, PS─Fe@TA‐Zr exhibited significantly higher Pb(II) removal efficiency, with a capacity of ≈58.88 mg g^−1^—nearly three times that of PS─Fe. In addition, the adsorption capacity of PS@TA‐Zr (15.00 mg g^−1^) is close to that of PS─Fe (17.93 mg g^−1^). This notable enhancement is attributed to the optimized utilization of HFO nanoparticles facilitated by the TA‐Zr (MPN) confinement. The cross‐linked confined HFO structure and strong electronegativity of TA promote the diffusion of Pb(II) ions into the inner spaces of the HFO nanoparticles, thereby maximizing the efficiency of Fe atom utilization. Consequently, this synergy significantly boosts the adsorption performance of the PS─Fe@TA‐Zr composite, the detailed verification is elucidated by the following section through XPS ion sputtering and DFT calculation.

To further quantify the selective adsorption capacity of PS─Fe and PS─Fe@TA‐Zr for Pb(II), the distribution coefficient (K_d_) of both adsorbents for Pb(II) in a competitive system was calculated.^[^ ^11^ ^]^

(1) $$K_{d}=\frac{\left(C_{0}-C_{e}\right)}{C_{e}}\frac{V}{m}$$

where C_0_ denotes the initial Pb(II) concentration, C_e_ represents the residual Pb(II) concentration after adsorption, V signifies the volume of the solution, and m indicates the mass of the adsorbent utilized. Despite increasing concentrations of competing ions, the K_d_ values for both PS─Fe and PS─Fe@TA‐Zr remained stable (Figure 2g–i). Notably, at high Ca(II) concentrations, PS─Fe@TA‐Zr achieved a K_d_ of 15278 mL g^−1^ approximately eight times higher than that of PS─Fe‐demonstrating its superior selectivity and enhanced HFO utilization for Pb(II) removal (Figure 2g). Similar enhancements were observed with Mg(II) and Na(I) ions (Figure 2h,i). Moreover, the influence of anions such as common chloride and sulfate was not considered in this study, as the primary competition for heavy metals occurs at cationic sites, rendering the impact of anions comparatively negligible.

Furthermore, we investigated the selectivity of PS─Fe@TA‐Zr toward Pb(II) in the presence of coexisting diverse heavy metals. As depicted in Figure S3a–c (Supporting Information), the presence of high concentrations of Co(II), Ni(II), and Cu(II) have a negligible impact on the adsorption capacity, indicating the high selectivity of PS─Fe@TA‐Zr. In particular, when the ratio of M(II) to Pb(II) reached 60 (M representing Co, Ni, or Cu), the PS─Fe@TA‐Zr maintained a Pb(II) adsorption capacity of 58.88 mg g^−1^, by contrast, the adsorption capacity for M(II) significantly drops to less than ≈10 mg g^−1^. This observation highlights the unique adsorption selectivity and strong affinity exhibited by PS─Fe@TA‐Zr.

#### 2.3.2 Fe Utilization Verification and Different Coordination Mechanism

**Figure 3:**
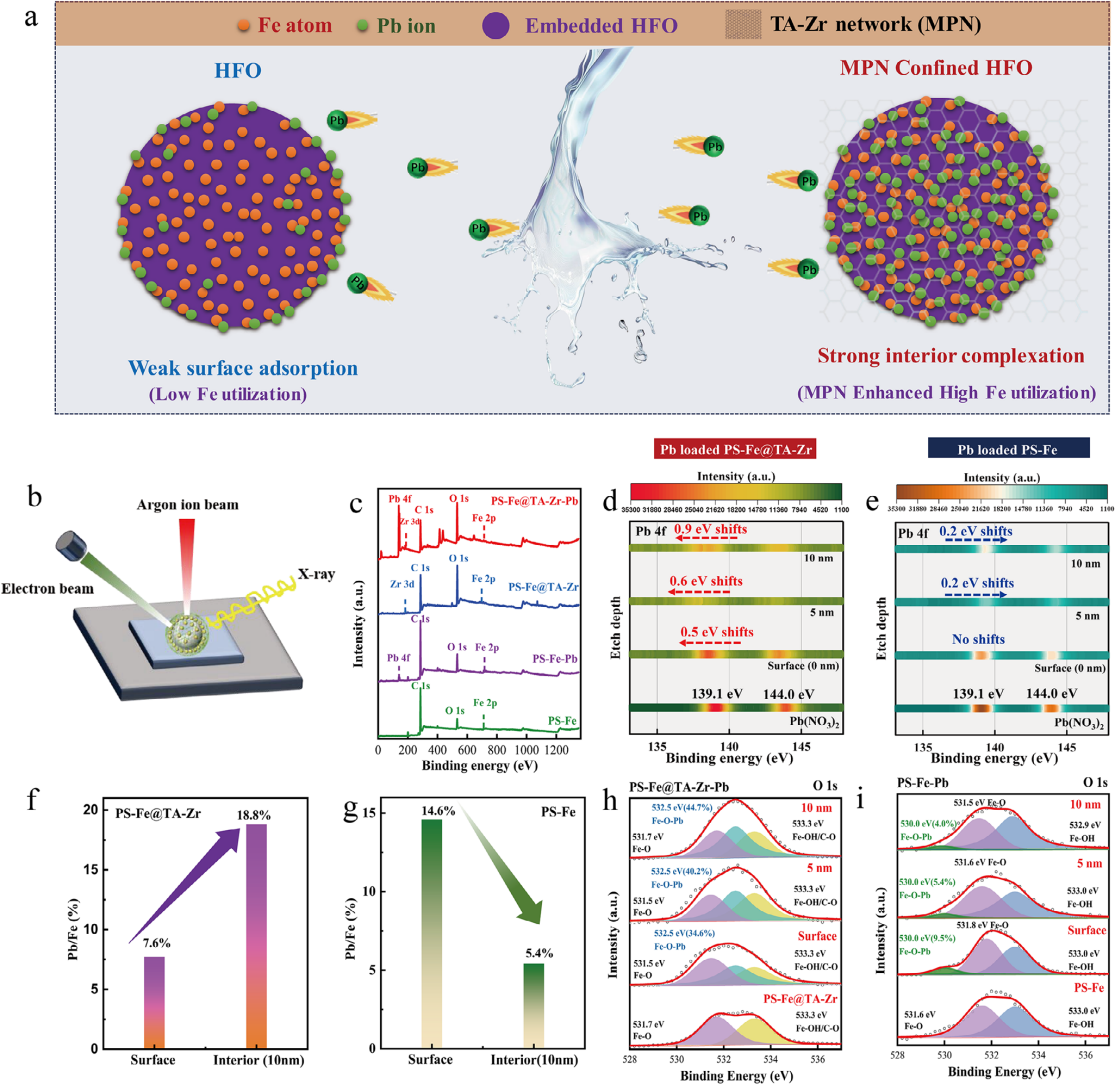
a) The comparison of Fe utilization rates of PS─Fe and PS─Fe@TA‐Zr. b)XPS etching of PS─Fe@TA‐Zr. c) XPS spectra of PS─Fe, PS─Fe@TA‐Zr and Pb(II) uptake samples. d,e) Pb(II) binding energies changes of PS─Fe@TA‐Zr and PS─Fe at different etching depths. f,g) Pb/Fe ratio of PS─Fe@TA‐Zr and PS─Fe at the surface and interior (10 nm). h,i) O 1 s spectra of PS─Fe@TA‐Zr and PS─Fe at different etching depths.

**Figure** **3**a illustrates the comparison of Fe utilization rates of PS─Fe and PS─Fe@TA‐Zr in the Pb(II) adsorption process. Furthermore, the enhanced Fe utilization was confirmed through XPS analysis and in‐depth ion sputtering investigation (Figure 3b). Figure 3c presents the survey scan spectrum, revealing the presence of C 1s, O 1s, Zr 3d, and Fe 2p peaks, indicative of the TA‐Zr network and the confined HFO nanoparticles. The pronounced Pb 4d and Pb 4f peaks confirm the successful Pb(II) scavenging by the PS─Fe@TA‐Zr composite.

Argon ion sputtering experiments unveiled the progressive evolution of the electronic structure of the samples as the etching depth varied from 0 to 10 nm, shedding light on the unique interaction between PS─Fe@TA‐Zr and Pb(II) as well as a detailed assessment of the utilization efficiency within the nano‐HFO. In this section, the Pb 4f scan spectra of Pb(NO₃)₂ were utilized as the standard reference sample for further comparative analysis. Figure 3d,e reveal that the deconvoluted peaks for Pb(NO₃)₂ are centered at 144.0 and 139.1 eV, corresponding to Pb 4f₅/₂ and Pb 4f₇/₂, respectively.^[^ ^3b^ ^]^ For the reacted PS─Fe, the intensity of Pb 4f peak sharply declined with increasing the etching depths, indicating that Pb(II) capture by PS─Fe predominantly relies on surface adsorption. Moreover, the slight shift (≈0.2 eV) to the higher binding energy of Pb 4f further suggested a negligible variation in the electronic structure of trapped Pb(II) with a weakened outer‐sphere complexation between Pb(II) and the HFO nanoparticles (Figure 3e). In contrast, Pb(II) adsorption onto PS─Fe@TA‐Zr, a strong Pb 4f signal was still detected even as the etching depth increased to 10 nm, indicating the presence of numerous adsorption sites within the internal structure of PS─Fe@TA‐Zr. Furthermore, a significant shift of 0.5–0.9 eV to lower binding energy was observed. The binding energy shift within the inner layers (≈0.9 eV. 10 nm) was substantially higher than that at the surface (≈0.5 eV. 0 nm) (Figure 3d), indicating more stable Fe─O─Pb coordination bonds formation and higher Fe utilization in the internal regions of the PS─Fe@TA‐Zr composite, due to the TA‐Zr confinement by favorable electronic structure arrangement.

Moreover, we conducted a detailed investigation of the variation in Fe utilization efficiency with increasing etching depth, revealing a significant rise in the Pb/Fe ratio (from 7.6% at the surface to 18.8% at a depth of 10 nm) (Figure 3f). This suggests that a substantial proportion of Pb(II) is adsorbed within the internal atomic structure of the HFO nanoparticles, rather than being confined to surface adsorption, highlighting the essential role of optimizing Fe utilization efficiency by TA‐Zr confinement in the adsorption process. The increase of Fe utilization was evident in the adsorption capacity, from 82.0 mg g^−1^ of PS─Fe to 222.7 mg g^−1^ of PS─Fe@TA‐Zr (Figure S5a, Supporting Information), almost three times the previous adsorption capacity. On the other hand, in terms of cost‐effectiveness, the estimated cost of treating per ton of Pb(II)‐wastewater with PS─Fe@TA‐Zr is 0.28 USD, which is much lower than that of similar adsorption materials (0.6–0.8 USD), detailed cost analysis is provided in Section 2.5. In contrast, Pb(II) adsorption on PS─Fe primarily occurs at the surface, with a notable decrease in Pb/Fe content within the inner layers of the HFO nanoparticles (from 14.6% at the surface to 5.4% at a depth of 10 nm) (Figure 3g).

To elucidate the specific interaction sites involved in the strong Pb(II) complexation, a comparative investigation of the O 1s XPS spectra for both fresh PS─Fe@TA‐Zr and Pb(II)‐adsorbed samples was performed.

The O 1s spectrum of PS─Fe@TA‐Zr is deconvoluted into two peaks at 531.7 and 533.3 eV, corresponding to Fe─O and Fe─OH/C─O bonds,^[^ ^29^ ^]^ respectively (Figure 3i). After Pb(II) adsorption, a new peak appears at 532.5 eV, along with a marked reduction in the Fe─O and Fe─OH peak areas. This new peak, with a band shift of ≈0.8 eV, represents electron redistribution at the O sites, likely resulting from the formation of a robust Fe─O─Pb complex, thereby confirming the strong adsorption affinity of Pb(II). Furthermore, the distribution of the emerging Fe─O─Pb peaks and their area ratios increase from 34.6% at the surface (0 nm) to 44.7% at an etching depth of 10 nm, further demonstrating enhanced Fe utilization and indicating that the strong Fe─O─Pb coordination interactions occur predominantly within the inner atomic layers

In contrast, after the uptake of Pb(II) by PS─Fe, the peak position of the Fe─O─Pb bond shifts to a lower binding energy of 530.0 eV (Figure 3g). Notably, the proportion of Fe─O─Pb bonds in PS‐Fe is only 9.5%, and this decreases significantly to 4.0% in the subsurface layer (10 nm in‐depth). This reduction can be attributed to the fact that the primary driving force in PS─Fe is outer‐sphere complexation. Pb is predominantly removed through coordination with surface Fe atoms, while the contribution of inner‐layer atoms is relatively negligible. Furthermore, we observed that the Fe─O─Pb complex formed on PS─Fe exhibits a binding energy at 530.0 eV, whereas the TA‐Zr network leads to a distinct binding energy shift for Fe─O─Pb at 532.5 eV. These significant variations further confirm the different driving forces and electronic structure arrangement between HFO and Pb(II), which are attributed to variations in electron cloud density and highly negative environment influenced by the TA‐Zr network.

The adsorption of Pb(II) in PS─Fe@TA‐Zr induces a shift in the O 1s binding energy to higher values, indicating a reduction in electron density around the O sites due to electron transfer from the TA‐Zr network to Pb(II). According to molecular orbital theory, the TA‐Zr‐anchored O site causes a downward shift in the O 1s orbital energy level, enhancing the alignment with Pb 4f orbital energy levels. This promotes the formation of robust Fe─O─Pb interactions. These findings suggest The adsorption of Pb(II) onto PS─Fe@TA‐Zr is primarily governed by inner‐sphere complexation alongside electrostatic enrichment effects. While the TA‐Zr confined nano HFO enhances charge enrichment in the subsurface layer, facilitating Pb(II) removal and optimizing Fe utilization. The TA‐Zr confinement also modifies the electronic structure and density of Fe and O sites, resulting in stronger interactions with Pb(II) in the subsurface layers.

**Figure 4:**
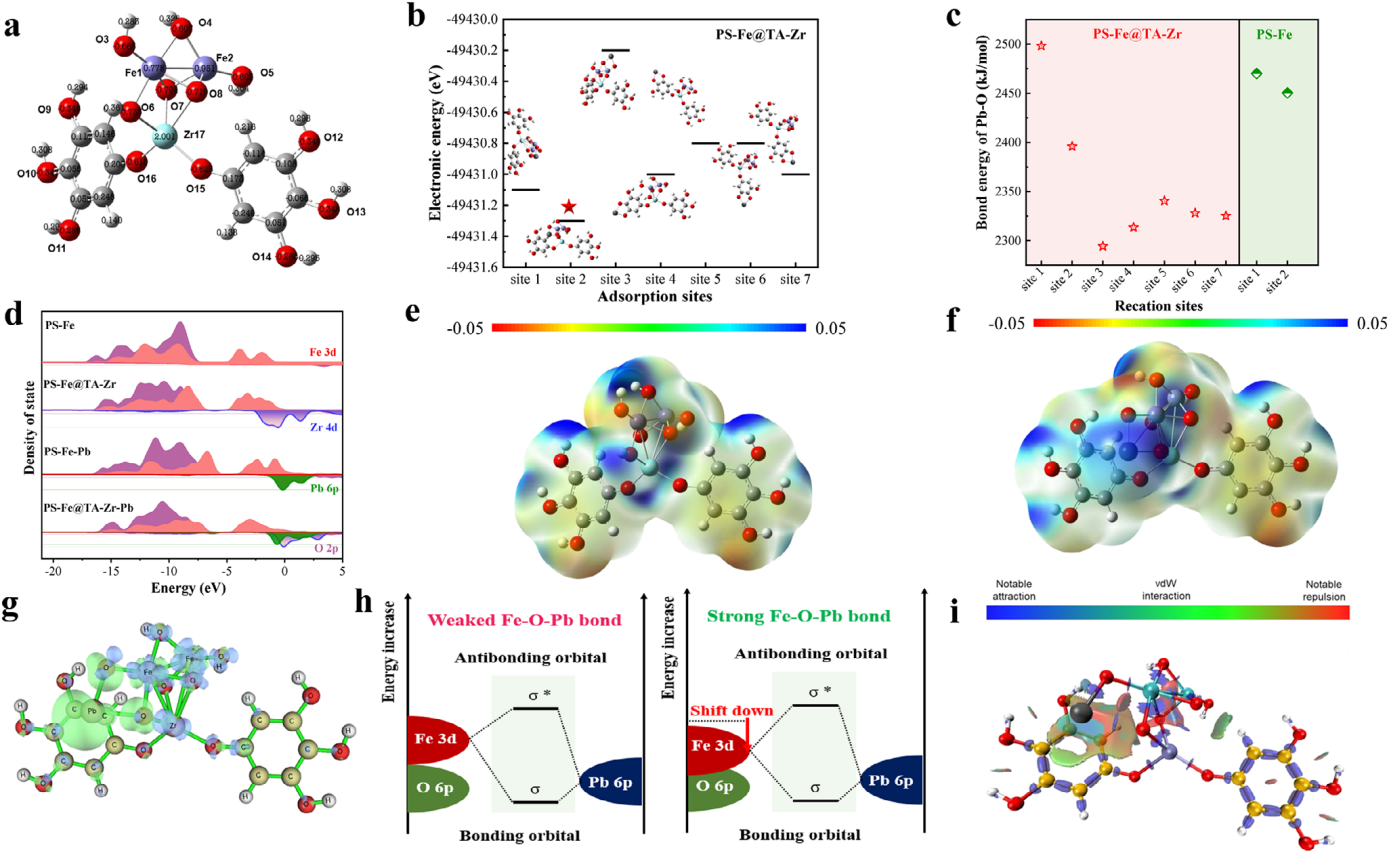
a) The optimal structure of PS─Fe@TA‐Zr calculated by DFT. b) The comparison of electronic energy of different adsorption site on PS─Fe@TA‐Zr. c) The comparison of bond energy of Pb‐O of different adsorption site in both PS─Fe@TA‐Zr and PS─Fe. d) PDOS analysis of PS─Fe, PS─Fe@TA‐Zr and Pb(II) uptake samples. e,f) The electrostatic potential (ESP) distributions of PS─Fe@TA‐Zr before and after adsorbing Pb(II). g) The differential charge density analysis of PS─Fe@TA‐Zr (green: electron density accumulation, blue: electron density depletion). h) Molecular orbital interaction diagram of Pb(II) adsorption of PS─Fe and PS─Fe@TA‐Zr. i) The interaction region indicator (IRI) of adsorption structure of PS─Fe@TA‐Zr.

Density functional theory (DFT) calculations were performed to explore the electronic structure, unique interactions, and active centers of the nanocomposites relevant to efficient Pb(II) adsorption. This comprehensive analysis included theoretical geometric and electronic properties, electrostatic potential distributions, density‐of‐states analysis, and electron density difference mapping. The finite‐size α‐Fe_2_O_3_ structure including two Fe elements, six O elements, and six H elements was modeled as the surface of PS─Fe to simplify the calculation (Figure S4, Supporting Information),^[^ ^30^ ^]^ also, Polyhydroxyphenol was used as a simple model of the TA structure.^[^ ^31^ ^]^ According to the preparation method, TA and Zr(IV) are initially complexed to form the TA‐Zr network, which is subsequently connected to PS─Fe through oxygen bridges, resulting in the formation of PS─Fe@TA‐Zr. The optimized model of PS─Fe@TA‐Zr is shown in **Figure** **4**a. The Pb adsorption sites of PS─Fe@TA‐Zr were calculated using DFT, revealing the exposure of seven active adsorption sites within PS─Fe@TA‐Zr. Sites 1 and 2 belong to the binding sites of Fe─O and Pb on PS─Fe@TA‐Zr, and sites 3–7 are the binding sites of oxygen and Pb on the TA‐Zr network (Figure 4b). Among all active sites on PS─Fe@TA‐Zr, site 2 has the lowest electronic energy (−49431.3 eV), suggesting that Fe─O─Pb forms a more stable structure, which means that Fe─O plays a major role in the adsorption of Pb(II), rather than the oxygen site on the TA‐Zr network. This significantly exceeds the two active sites observed in PS─Fe without TA‐Zr confinement, which corresponds with the results of XPS analysis. (Figure 4b; Figure S5, Supporting Information). In terms of Pb─O bond energy, the bonding energy of Fe─O for PS‐Fe@TA‐Zr (sites 1 and 2) to Pb(II) is much higher than that of TA‐Zr network (sites 3–7). This further confirms that Pb(II) is preferentially bound to oxygen sites on Fe─O rather than TA‐Zr network (Figure 4c). In addition, the highest bond energy of Pb‐O bond for PS‐Fe@TA‐Zr (≈2498.2 kJ mol^−1^) is significantly higher than that of PS─Fe (≈2470.2 kJ mol^−1^), indicating that the coordination structure formed between PS─Fe@TA‐Zr and Pb is thermodynamically more stable than that of PS─Fe (Figure 4c). It is important to highlight that the Pb(II) ions coordinated by O3 and O6 in the surface model of PS─Fe@TA‐Zr exhibit the most stable configuration, as indicated by their lowest electronic energy. Consequently, the O sites with lower charges that are bonded to Fe emerge as the preferred adsorption sites for Pb(II) among the seven identified sites.

Figure 4d illustrates the partial density of states (PDOS) of Fe 3d, O 2p, Zr 4d, and Pb 6p on the surface of PS─Fe and PS─Fe@TA‐Zr after Pb(II) adsorption. In PS─Fe, the overlapping bands of Fe 3d and O 2p are observed within the energy range of −3.75–−0.12 eV. Conversely, these bands shift to lower energies, spanning −4.50–−1.20 eV in PS─Fe@TA‐Zr, indicating the formation of a more stable Fe─O─Pb bond at the O sites. We then calculated the electrostatic potential (ESP) distribution of PS─Fe@TA‐Zr before and after Pb(II) adsorption (Figure 4e). Initially, the positive electrostatic potential was predominantly localized around the Fe site. However, after Pb(II) adsorption, significant changes occurred in the ESP (Figure 4f), specifically, the area surrounding the TA‐Zr network transitioned to a negative electrostatic region while a larger area near the Pb(II) site exhibited a positive potential. This indicates the electron donor role of TA‐Zr network on PS─Fe@TA‐Zr, namely the ability to provide electrons, plays an important role in enhancing Pb(II) adsorption. Furthermore, the differential charge density for PS─Fe@TA‐Zr derived from DFT calculation facilitates visualization of the variations in electron cloud density at the O sites (Figure 4g). Notably, the bonding region of Fe─O─Zr in PS‐Fe@TA‐Zr appears as an electron density depletion zone around the O sites, while the Pb─O interaction exhibits an electron density accumulation region. This observation indicates that electrons are predominantly transferred from Fe/Zr to Pb(II) via the oxygen bridge, significantly enhancing the adsorption of Pb(II) by the TA‐Zr‐confined nano‐HFO. According to molecular orbital theory, the efficiency of bonding orbital formation is enhanced when the energy levels of the atomic orbitals involved in molecular orbital creation are closely aligned. In the case of the TA‐Zr network, the anchored O sites induce a downward shift in the energy level of the Fe 3d orbitals, attributed to a reduction in electron density (Figure 4h).^[^ ^32^ ^]^ This shift facilitates a more favorable alignment between the Fe 3d and Pb 6p orbital energy levels, thereby significantly promoting the formation of bonding orbitals. Consequently, robust Fe─O─Pb interactions are established, leading to the highly efficient removal of Pb(II) ions. The Interaction Region Indicator (IRI) was calculated to elucidate the interactions between the PS‐Fe@TA‐Zr surface and Pb(II) ions. The IRI iso‐surface is represented using a blue‐green‐red scale, where blue, green, and red disks correspond to chemical bonds, van der Waals forces, and repulsion, respectively. In Figure 4i, it is evident that the adsorption of Pb(II) at the preferred sites is predominantly governed by complexation with O sites, and the electrostatic attraction arising from delocalized electrons on the benzene ring.

### 2.4 The Pb(II) Diffusion and Enrichment by Charged MPN Driven

**Figure 5:**
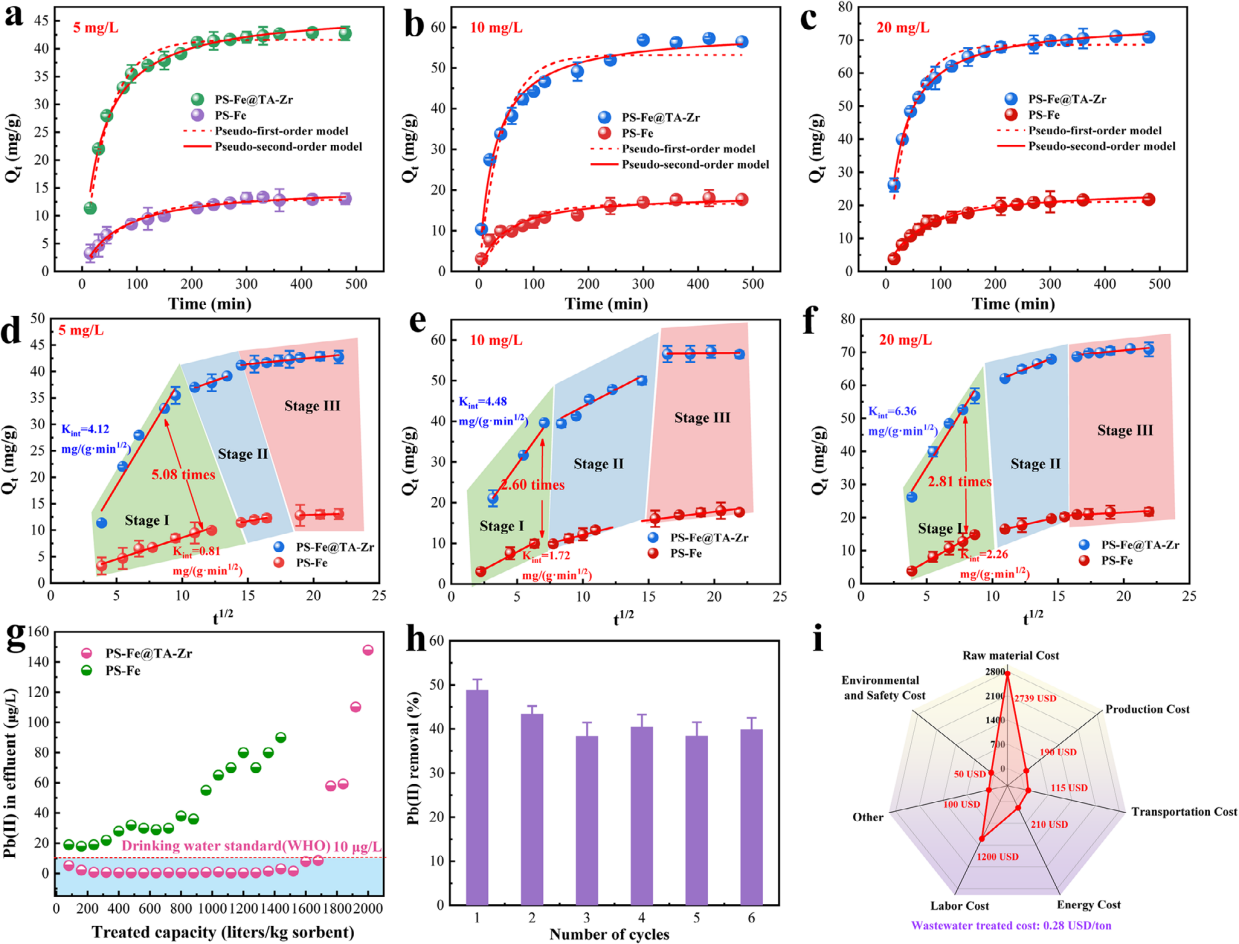
a–c) Pb(II) sorption kinetic curves of the PS─Fe and PS─Fe@TA‐Zr at an initial concentration of 5, 10, and 20 mg L^−1^. d–f) Intraparticle diffusion model of Pb(II); Conditions: 0.1 g L^−1^ absorbents, 500 mL solution, 298 K for 10 h reaction, pH = 5.6. g) Continuous adsorption capacity assessment of PS─Fe and PS─Fe@TA‐Zr on Pb(II)‐contaminated wastewater, detail parameters are shown in Table S4, pH = 4.00. h) Cycles Pb(II) removal by PS─Fe@TA‐Zr. i) Comprehensive cost estimation for PS─Fe@TA‐Zr heavy metal wastewater purification system.

The utilization of nano‐HFO is closely associated with the diffusion and kinetic behaviors of heavy metals, facilitated by the strong electronegativity of MPN. The kinetic behaviors were conducted to examine the diffusion behavior of Pb(II) by PS─Fe@TA‐Zr at varying concentrations. The pseudo‐first‐order, pseudo‐second‐order,^[^ ^33^ ^]^ and intra‐particle diffusion models^[^ ^3b^ ^]^ were used to analyze sorption behaviors, with fitting curves and parameters shown in **Figure** **5**a–f and Table S2 (Supporting Information).

For the removal of trace Pb(II) at ≈5 mg L^−1^, PS─Fe@TA‐Zr reached adsorption equilibrium in ≈150 min, whereas PS─Fe required ≈300 min. This also resulted in over a fourfold increase in Pb removal capacity for PS─Fe@TA‐Zr. The sorption behaviors were effectively described by the pseudo‐second‐order kinetic model, demonstrating a high correlation coefficient (Figure 5a–c; Table S2, Supporting Information). To quantify the kinetic process, we compared the intraparticle diffusion coefficient, the rate constant K_int_ (k_1_ = 4.12) in stage I of PS─Fe@TA‐Zr is almost 5 times larger than that of PS─Fe (k_1_^’^ = 0.81), further demonstrating the diffusion enhancement by TA‐Zr (MPN) confinement (Figure 5d). The significant increase in mass transfer rate can be attributed to the charged MPN accelerating Pb diffusion and enrichment around nano‐HFO, as well as variations in electron density and Pb 4f orbital alignment induced by the TA‐Zr (MPN). Simultaneously, the equilibrium adsorption capacity of PS─Fe@TA‐Zr was 42.7 mg g^−1^, compared to only 13.1 mg g^−1^ for PS─Fe. This result confirms the remarkable improvement in Fe utilization driven by the TA‐Zr (MPN) networks. As the initial Pb(II) concentration increased to 10 and 20 mg L^−1^, the liquid film mass transfer rate significantly accelerated. Despite this, the rate constant and adsorption capacity of PS─Fe remained significantly inferior compared to those of PS─Fe@TA‐Zr (Figure 5e,f). This highlights the consistently superior enhancement in diffusion and permeation driven by the MPN network confinement, demonstrating its strong and concentration‐independent driving force.

To assess the enhancement effect of the TA‐Zr network, the adsorption capacities of PS─Fe, PS@TA‐Zr, and PS─Fe@TA‐Zr were evaluated through isotherm studies. As illustrated in Figure S6a (Supporting Information), PS@TA‐Zr demonstrated a markedly lower Pb(II) adsorption capacity (23.0 mg g^−1^) compared to PS─Fe (82.0 mg g^−1^) and PS─Fe@TA‐Zr (222.7 mg g^−1^), thereby affirming the synergistic effect of the TA‐Zr (MPN) network confined nano‐HFO. Additionally, the adsorption capacity improved at lower temperatures (Figure S6b,c, Supporting Information), indicating an exothermic nature of the adsorption process. The Freundlich model,^[^ ^34^ ^]^ with a high correlation coefficient (R^2^ > 0.98), effectively describes this adsorption process, reflecting the strong affinity of heterogeneous surfaces for Pb(II) ions (Figure S6c, Supporting Information).

### 2.5 Real Application Verification and Economic Evaluation

Fixed‐bed column experiments were conducted to assess the practical applicability of the PS─Fe@TA‐Zr nanocomposite for the treatment of real wastewater. The Pb(II)‐contaminated wastewater, sourced from an electroplating facility in Qinhuangdao City, was analyzed, with water quality parameters detailed in Table S4 (Supporting Information). The breakthrough point for Pb(II) was established at 10 µg L^−1^, in accordance with WHO drinking water standards. The results demonstrate that the PS─Fe@TA‐Zr nanocomposite exhibits exceptional performance in Pb(II) removal from real water streams, achieving a treatment capacity of 1680 liters of wastewater per kilogram of adsorbent (Figure 5g). Notably, the implementation of this technology enables the treatment of industrial wastewater to meet potable water standards, a level that PS─Fe materials cannot attain. The leaching of TA, Fe, and Zr during the experiment was also monitored at different intervals (Figure S7, Supporting Information). In the pre‐adsorption phase (0–500 liters kg^−1^ sorbent), there is a small loss of PS─Fe@TA‐Zr nanocomposite components (TA, Fe, and Zr) (<0.2 mg L^−1^) due to surface deposition of the material. As the adsorption proceeds, the loss of material is negligible, verifying the environmental safety and stability of the PS─Fe@TA‐Zr nanocomposites.

Furthermore, a comprehensive economic analysis was performed to assess the commercial viability of the PS─Fe@TA‐Zr nanocomposite for large‐scale industrial applications (Table S5, Supporting Information). Also, producing TA from organic agricultural waste provided a feasible pathway to reduce costs.^[^ ^21^ ^]^ Our assessment starts with a breakdown of the one‐time investments required for equipment: PS─Fe@TA‐Zr synthesis equipment ($11000), auxiliary systems such as oscillators and pipes ($5000), wastewater disinfection system ($35000) and other equipment ($6000), for a total of $57000. The system is capable of producing at least 10 tons PS─Fe@TA‐Zr per batch and treating up to 16800 tons of wastewater, with treatment capacity based on data from Figure 5g. Next, we evaluated the operational costs. The raw material cost for PS─Fe@TA‐Zr is ≈$2739 per batch. The energy cost (such as electricity) of treating 1 ton of water is $0.0125, and the total electricity bill is $210. Additionally, considering transportation costs ($115), labor costs ($1200), production costs ($190), environmental and safety costs ($50), and other overheads ($100), the final treatment cost per ton of wastewater is estimated at 0.28 USD per ton for Pb(II) removal, while the average cost (such as IRC‐748, an iminodiacetic acid chelated cation exchange resin) of wastewater treatment ranges from 0.6 to 0.8 USD per ton (Figure 5i). These results highlight that PS─Fe@TA‐Zr offers a highly cost‐effective solution for the treatment of Pb(II)‐contaminating wastewater.

The PS─Fe@TA‐Zr nanocomposite can be regenerated through acid treatment. The saturated PS─Fe@TA‐Zr underwent in situ regeneration with a 0.005% HNO_3_ solution. Over six continuous adsorption‐desorption cycles, the removal performance of Pb(II) by PS─Fe@TA‐Zr remained stable, indicating the nanocomposite's excellent reusability and high stability across multiple cycles (Figure 5h). The regeneration mechanism of PS─Fe@TA‐Zr material is attributed to the hydroxyl protonation of HFO and pH‐responsive coordinated structure of MPNs in an acidic solution. At low pH, the TA‐Zr network changes from a stable six‐coordination structure to a weaker two‐coordination configuration, releasing a small amount of Pb(II). At the same time, the hydroxyl group on HFO was protonated under acidic conditions, the original active site of HFO was restored, and most of the remaining Pb(II) was released, achieving PS─Fe@TA‐Zr regeneration.

## 3 Conclusion

In recent years, iron‐based nanocomposites have been extensively developed and applied for the removal of heavy metals. However, the low utilization of nanoparticles, such as HFO, remains a critical challenge, often overlooked, leading to substantial losses in practical application efficiency. In this study, we introduced an innovative metal‐phenolic network (TA‐Zr, MPN) strategy to optimize iron utilization through the in situ synthesis of TA‐Zr‐confined nano‐HFO within polystyrene beads (PS─Fe@TA‐Zr). This approach induces a negative electrostatic field and precisely regulates the electron density at oxygen coordination sites, thereby significantly enhancing nano‐iron utilization and greatly improving its efficiency in the deep purification of heavy metals. Our findings reveal that the TA‐Zr‐mediated nanocomposite facilitates charge enrichment of heavy metals and enhances Pb(II) removal while optimizing Fe utilization. Notably, the Pb/Fe content increased substantially from 7.6% at the surface to 18.8% at an etching interior depth of 10 nm, In contrast, conventional nanocomposites, such as PS─Fe, the nano‐HFO, primarily remove Pb(II) through surface adsorption. Furthermore, PS─Fe@TA‐Zr demonstrates selectivity that is over 8 times greater in high‐salinity environments, achieving an exceptional K value of 15278 mL g^−1^; it also exhibits rapid kinetics, with a kinetic rate (K_int_) 5 times greater than that of PS─Fe, and achieves a treatment capacity of 1680 liters per kilogram while maintaining effective generation for up to 6 cycles in actual industrial wastewater.

## 4 Experimental Section

### Materials

All chemical reagents used in this study were of analytical grade and required no further purification. Tannic acid (TA), iron(III) chloride (FeCl_3_), zirconium oxychloride octahydrate (ZrOCl_2_·8H_2_O), lead nitrate (Pb(NO_3_)_2_), and 3‐morpholinopropanesulfonic acid (MOPs) were sourced from Shanghai Meryer Chemical Technology Co. LTD, China. The polystyrene resin (PS) employed in this research as a matrix was generously provided by Zhengguang Resin Co, Hangzhou, China. Prior to its utilization, the resin underwent a purification step involving washing with 90% ethanol to eliminate impurities.

### Fabrication of PS─Fe@TA‐Zr—Synthesis of Fe‐Encapsulated Nanocomposite (PS─Fe)

The Fe‐encapsulated nanocomposite (PS─Fe) was synthesized using an in situ deposition method. To outline the procedure briefly, 16.8 g of FeCl_3_ was dissolved in 220 mL of a 90% ethanol solution. Next, 4.0 g of PS was introduced into the solution at 50 °C and allowed to react for 16 h, facilitating the diffusion of Fe ions into the porous structure of PS. Subsequently, the resulting mixture was combined with 200 mL of a 1% NaOH solution and stirred for 12 h, allowing for a gradual deposition process. Following this, the Fe‐encapsulated nanocomposite (PS─Fe) material was thoroughly washed to achieve a neutral environment and then dried for subsequent applications.

### Fabrication of PS─Fe@TA‐Zr—Preparation of the Targeted PS─Fe@TA‐Zr Nanocomposite

To prepare the desired PS─Fe@TA‐Zr nanocomposite, 2.0 g of PS─Fe was introduced into a 100 mL solution containing 200 mm MOPs as a buffer with a pH of 8.5. Subsequently, 0.4 g of TA and 0.15 g of ZrOCl_2_·8H_2_O were meticulously dissolved and stirred for a duration of 1 h, resulting in the formation of the TA‐Zr network. The resulting PS─Fe@TA‐Zr nanocomposite was then thoroughly rinsed with deionized water and subsequently dried, making it ready for further applications.

### Batch Adsorption Experiments

Batch adsorption experiments were carried out using conventional bottle‐point methods. The experimental procedure is outlined as follows:

0.15 g of PS─Fe@TA‐Zr adsorbents were introduced into bottles, each containing 50 mL of a 10 mg L^−1^ Pb(II) solution. PS─Fe was also included as control groups. The pH of the solution was carefully adjusted within the desired range (1.0–7.0) by employing 1% NaOH and 1% HCl. Subsequently, the samples were subjected to agitation at 180 rpm for a duration of 12 h at a temperature of 298 K, utilizing an incubator shaker (SHZ85 JINTAN, China). The release of Fe and Zr was quantified using ICP‐MS (Inductively Coupled Plasma Mass Spectrometry).

The investigation delved into the impact of co‐existing ions, encompassing the abundant ions such as Ca(II), Mg(II), and Na(I) at elevated concentrations. To establish a robust benchmark, PS─Fe was employed as reference materials for comparative analysis. Moreover, kinetic investigations were conducted by introducing 3.0 g of PS─Fe@TA‐Zr into a 500 mL aqueous solution containing Pb(II) at varying concentrations (Pb(II) = 5, 10, 20 mg L^−1^). The supernatant was collected at discrete time intervals to elucidate the adsorption kinetics, with PS─Fe serving as the reference material in this kinetic study.

Additionally, the maximal adsorption capacity of PS─Fe@TA‐Zr was determined by scrutinizing a range of Pb(II) solutions with concentrations spanning from 10 to 100 mg L^−1^, while operating at distinct temperatures (293, 313, and 333 K). Within this framework, the well‐established PS─Fe and PS@TA‐Zr were selected as model adsorbents for meticulous comparison.

### Real Application in Column Verification

For rigorous capacity assessments, an intricately designed glass column, measuring 10 mm in diameter and extending 150 mm in length, was employed as the experimental apparatus. Within this column, 3 mL of the hydrated PS─Fe@TA‐Zr or PS─Fe adsorbent material was expertly packed. The influent, originating directly from an electroplating wastewater workshop in Qinhuangdao City, introduced an element of authenticity to the experimental setup. Detailed operational parameters have been comprehensively cataloged in Table S4 (Supporting Information) for scholarly reference.

To ensure precise regulation of the flow rate at a consistent 10 bed volume per hour (BV/h), an HL‐2D pump from Shanghai, China, was integrated into the experimental system. An automated fraction collector was also employed to efficiently gather effluent samples, resulting in a well‐coordinated experimental setup. This advanced configuration enabled the evaluation of effluent Pb(II) concentration, facilitating the construction of a curve that illustrates the relationship between treatment capacity and effluent concentration. The exhausted PS─Fe@TA‐Zr was regenerated using a 0.005% HNO_3_ solution for multi‐cycle evaluation.

### Analysis and Characterization

The PS─Fe@TA‐Zr underwent a comprehensive regime of characterization and analysis. The morphological features of the adsorbents were investigated using advanced techniques, including scanning electron microscopy (SEM, S4800‐II, Japan) and transmission electron microscopy (TEM, JEM2010, Japan). To gain insight into the structural properties, X‐ray diffraction (XRD) patterns were meticulously acquired using a powder diffractometer (XTRA, Switzerland), employing a step scan of 10° min^−1^ per step. Moreover, to probe the chemical compositions and functional groups, FTIR spectra of PS─Fe@TA‐Zr were meticulously obtained with a Fourier transform infrared spectrometer (Nexus 870, USA). Zeta potentials were thoughtfully measured utilizing photon correlation spectroscopy (Malvern Zetasizer 3000 HAS). To quantify the concentrations of various metal ions, two sophisticated techniques, namely atomic absorption spectrometry (AAS‐6800) and inductively coupled plasma emission spectrometry (ICP, PerkinElmer model Optima 5300DV, USA), were employed. Additionally, X‐ray photoelectron spectroscopy (XPS) analysis was undertaken using a state‐of‐the‐art spectrometer (ESCALAB‐2, Great Britain) for comprehensive surface analysis.

### Theoretical Calculations

The DFT calculations were performed with a Gaussian 16 software package, and the models were created using GaussView 6.0.^[^ ^35^ ^]^ The geometries optimization and frequency analyses of PS─Fe, PS─Fe@TA‐Zr, and PS─Fe@TA‐Zr‐Pb models were calculated under the double hybrid density functional method (PW6B95‐D3) with the Lanl2DZ pseudopotential and corresponding basis sets for transition metallic elements and the def‐TZVP basis set for other elements. The SMD solvation model was chosen to simulate the effect of water. The differential charge density, bond order analysis, DOS map, and IRI analysis of Models were calculated by Multiwfn software^[^ ^36^ ^]^ and VMD Program.^[^ ^37^ ^]^

## Conflict of Interest

The authors declare no conflict of interest.

## Author Contributions

M.Z. conceived and planned this research. X.D. and Z.L. performed the experiments. S.W. and Y.W. performed the XPS analysis and TEM images, N.C. and T.J. helped to provide the discussion in actual application performance, Y.S. discussed the XPS ion sputtering; K.S. performed the DFT calculation, Y.Y. and Q.Z. organized the data and wrote the manuscript. All authors discussed the results and approved the final version of the manuscript.

## Supporting information



Supporting Information

## Data Availability

Data sharing is not applicable to this article as no new data were created or analyzed in this study.
