# Supplementary material for: New Insights into Heavy Metal Sequestration Through Metal‐Phenolic Network‐Confined Nano‐HFO: Overlooking Iron Utilization and Modulating Electron Density
Source: Adv Sci (Weinh). 2025 Mar 28;12(22):2417798. doi: 10.1002/advs.202417798 (PMC12165113; doi:10.1002/advs.202417798)
Supplement: Supplementary file 1 — Supporting Information [file ADVS-12-2417798-s001.docx]

**Supporting Information**

**New Insights into Heavy Metal Sequestration through Metal-Phenolic Network-Confined Nano-HFO: Overlooking Iron Utilization and Modulating Electron Density**

Manyu Zhang^a^, Xiaolin Du^d^, Zhanqi Liu^a^, Yujia Yang^a^*, Shuo Wang^a^, Ningyi Chen^b^, Yulin Wang^a^, Yaran Song ^a^, Keju Sun ^a^, Qingrui Zhang^a, c^*

^a^ State Key Laboratory of Metastable Materials Science and Technology, Hebei Key Laboratory of Heavy Metal Deep-remediation in Water and Resource Reuse, Yanshan University Qinhuangdao 066004, China

^b^ College of Environment, Zhejiang University of Technology, Hangzhou, Zhejiang 310014, China

c Hebei Province Engineering Research Center for Harmless Synergistic Treatment and Recycling of Municipal Solid Waste, Yanshan University, Qinhuangdao, 066004, China

^d^ Hong Qi Sheng Precision Electronics (Qinhuangdao) Co., LTD.

**Text S1 Adsorption isotherm models**

Langmuir model:

$Q_{e}=\frac{Q_{max}K_{L}C_{e}}{1+K_{L}C_{e}}$ (S1)

Freundlich model:

$\lg Q_{e}=\lg K_{f}+\frac{1}{n}\lg C_{e}$ (S2)

Here, C_e_ represents the concentration of Pb (II) in equilibrium, Q_e_ reflects the corresponding adsorption capacity, Q_max_ is the maximum sorption capacity in calculation, K_F_ and K_L_ are adsorption coefficients. n is the Freundlich model coefficient.

**Text S2 Kinetic models**

Pseudo-first-order model:

$Q_{t}=Q_{e}\left( 1-e^{-k_{1}t} \right)$ (S4)

Pseudo-second-order model:

$\frac{t}{Q_{t}}=\frac{1}{k_{2}Q_{f}^{2}}+\frac{t}{Q_{f}}$ (S5)

Intraparticle diffusion model:

$Q_{t}=k_{int}t^{\frac{1}{2}}+\theta$ (S6)

Here, Q_t_ is the sorption capacity (mg/g) at different time intervals, and Q_e_ is the adsorption capacity in equilibrium; k_1_ (1/min) and k_2_ (g/(mg⋅min)) are the classic rate constants; k_int_ (mg/(g·min^1/2^)) represents the diffusion coefficient constant; and θ reflects the intercept of the intraparticle diffusion model.

**Table S1** The structure parameters of PS, PS-Fe and PS-Fe@TA-Zr.

| Samples | Specific surface area (m^2^ g^-1^) | Pore volume  (cm³ g^-1^) | Average pore size (nm) |
| --- | --- | --- | --- |
| PS | 34.86 | 0.267 | 30.64 |
| PS-Fe | 51.20 | 0.295 | 23.05 |
| PS-Fe@TA-Zr | 60.58 | 0.335 | 22.15 |

**Table S2** Kinetic fitting parameters of PS-Fe and PS-Fe@TA-Zr nanocomposite for Pb (II) removal

|  | **materials** | **Pseudo-first-order model** | | | | **Pseudo-second-order model** | | |
| --- | --- | --- | --- | --- | --- | --- | --- | --- |
|  |  | *Q_e_* (mg/L) | *K*_1_ (1/min) | *R*^2^ |  | *Q_e_* (mg/L) | *K*_2_ (g/(mg·min)) | *R*^2^ |
| 5 ppm | PS-Fe | 12.88 | 0.012 | 96.70 |  | 15.27 | 0.0009 | 98.68 |
|  | PS-Fe@TA-Zr | 41.60 | 0.022 | 98.10 |  | 46.86 | 0.0006 | 98.68 |
| 10 ppm | PS-Fe | 16.64 | 0.016 | 87.76 |  | 18.99 | 0.0012 | 99.46 |
|  | PS-Fe@TA-Zr | 53.21 | 0.024 | 88.03 |  | 59.14 | 0.0006 | 96.43 |
| 20 ppm | PS-Fe | 21.14 | 0.014 | 98.75 |  | 25.21 | 0.0007 | 99.27 |
|  | PS-Fe@TA-Zr | 68.56 | 0.026 | 96.73 |  | 2.541 | 0.0005 | 99.81 |

**Intraparticle diffusion model**

|  | **materials** | ***K*_int_** | | |  | ***R*^2^** | | |
| --- | --- | --- | --- | --- | --- | --- | --- | --- |
|  |  | **Ⅰ** | **Ⅱ** | **Ⅲ** |  | **Ⅰ** | **Ⅱ** | **Ⅲ** |
| 5 ppm | PS-Fe | 0.82 | 0.44 | 0.09 |  | 98.8 | 98.5 | 99.8 |
|  | PS-Fe@TA-Zr | 4.12 | 0.86 | 0.25 |  | 97.7 | 99.2 | 95.0 |
| 10 ppm | PS-Fe | 1.72 | 0.83 | 0.41 |  | 99.2 | 94.8 | 85.5 |
|  | PS-Fe@TA-Zr | 4.48 | 1.68 | 0.03 |  | 99.1 | 95.4 | 86.0 |
| 20 ppm | PS-Fe | 2.26 | 0.90 | 0.22 |  | 99.7 | 99.9 | 90.9 |
|  | PS-Fe@TA-Zr | 6.36 | 1.63 | 0.82 |  | 98.8 | 99.1 | 90.5 |

**Table S3** Isothermal fitting parameters of PS-Fe and PS-Fe@TA-Zr nanocomposite for Pb (II) removal

|  |  | **Langmuir** | | |  | **Freundlich** | | |
| --- | --- | --- | --- | --- | --- | --- | --- | --- |
| Tem(K) | materials | *Q_max_* (mg/g) | *K_L_*  (L/mg) | *R*^2^ |  | *K_F_* ((mg/g)/(mg/L)^n^) | *n* | *R*^2^ |
| 293 K | PS-Fe | 82.50 | 0.15 | 96.70 |  | 26.33 | 0.26 | 97.45 |
|  | PS-Fe@TA-Zr | 221.5 | 0.26 | 89.66 |  | 87.38 | 0.22 | 97.35 |
| 313 K | PS-Fe@TA-Zr | 201.0 | 0.15 | 95.18 |  | 57.14 | 0.29 | 98.55 |
| 333 K | PS-Fe@TA-Zr | 138.9 | 0.09 | 96.91 |  | 34.51 | 0.30 | 98.52 |

**Table S4** The real wastewater concentrations of each ion

| Ions | Pb^2+^ | Cd^2+^ | Ni^2+^ | Zn^2+^ | Ca^2+^ | Mg^2+^ | Na^+^ | Cl^-^ | NO_3_^-^ |
| --- | --- | --- | --- | --- | --- | --- | --- | --- | --- |
| Concentrations  (mg/L) | 1.23 | 4.39 | 9.20 | 16.00 | 100.00 | 188.00 | 635.00 | 257.00 | 358.00 |

**Table S5** The economic cost of synthetic and treatment of PS-Fe@TA-Zr

| **Cost Category** | **Description** | **Quantity/Frequency** | **Unit Cost (USD)** | **Total Cost (USD)** |
| --- | --- | --- | --- | --- |
| **Cost 10 tons of**  **PS-Fe@TA-Zr** | Synthesis equipment | 1 | 11,000 | 11,000 |
|  | Auxiliary systems | 1 | 5,000 | 5,000 |
|  | Wastewater disinfection system | 1 | 35,000 | 35,000 |
|  | Other equipment | - | - | 6,000 |
|  | **Total** | - | - | 57,000 |
| **Total Operational Costs** | Raw Material Cost  (PS-Fe@TA-Zr) | 0.55 tons/month | 4,980 | 2,739 |
|  | Production Cost | per month | - | 190 |
|  | Transportation Cost | per month | - | 115 |
|  | Energy Cost |  |  | 210 |
|  | Labor Cost | per month | - | 1,200 |
|  | Environmental and Safety Cost | per month | - | 50 |
|  | Other | - | - | 100 |
|  | **Total** | - | - | **4,604** |
| **Wastewater treated cost** | Heavy metal wastewater | 16,800 tons/month | 4,604 | **0.28** |


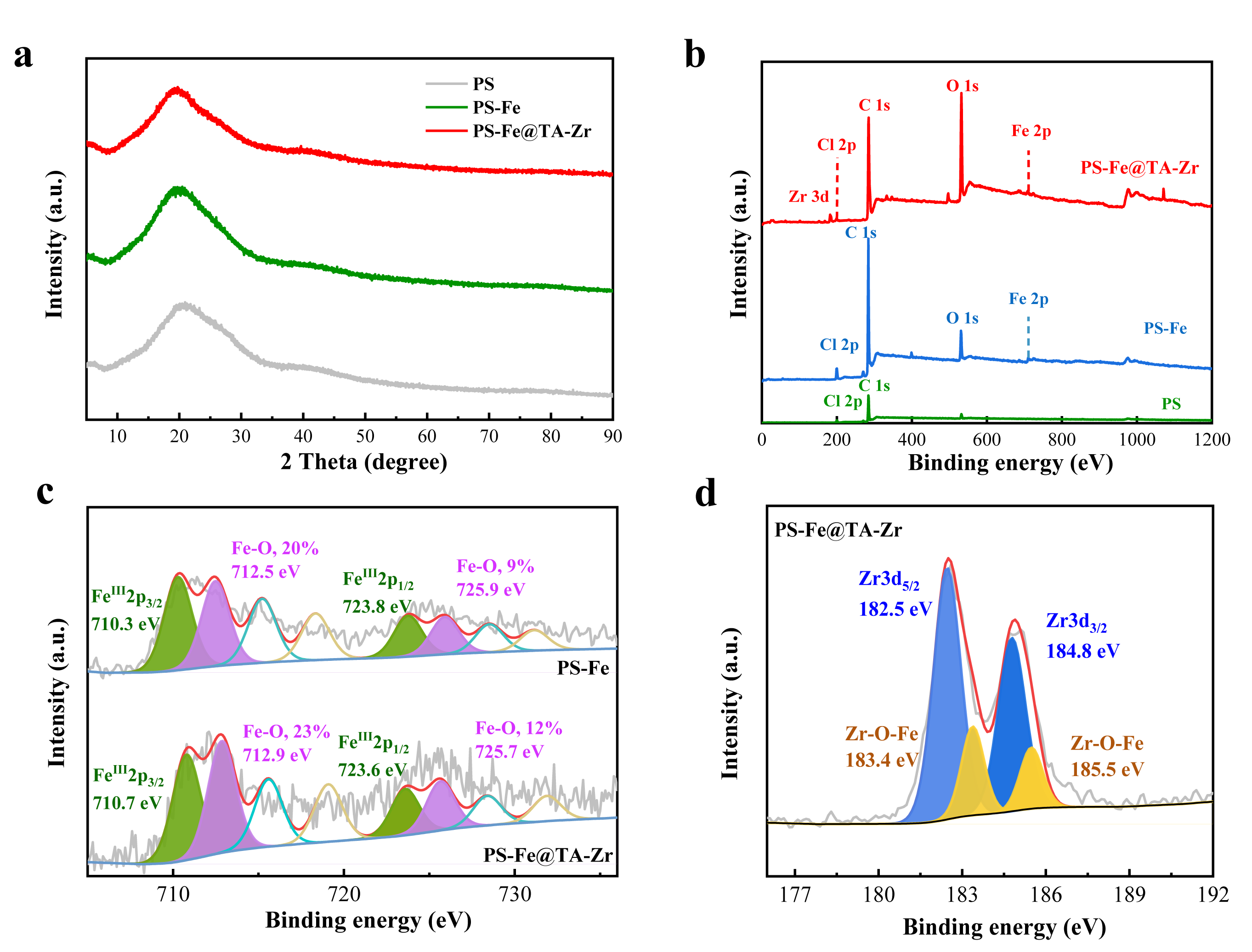


**Figure S1.** (a) XRD of PS, PS-Fe and PS-Fe@TA-Zr. (b) XPS spectra of PS, PS-Fe, and PS-Fe@TA-Zr. (c) Fe 2p spectra of PS-Fe, PS-Fe@TA-Zr. (d) Zr 4d spectra of PS-Fe@TA-Zr.


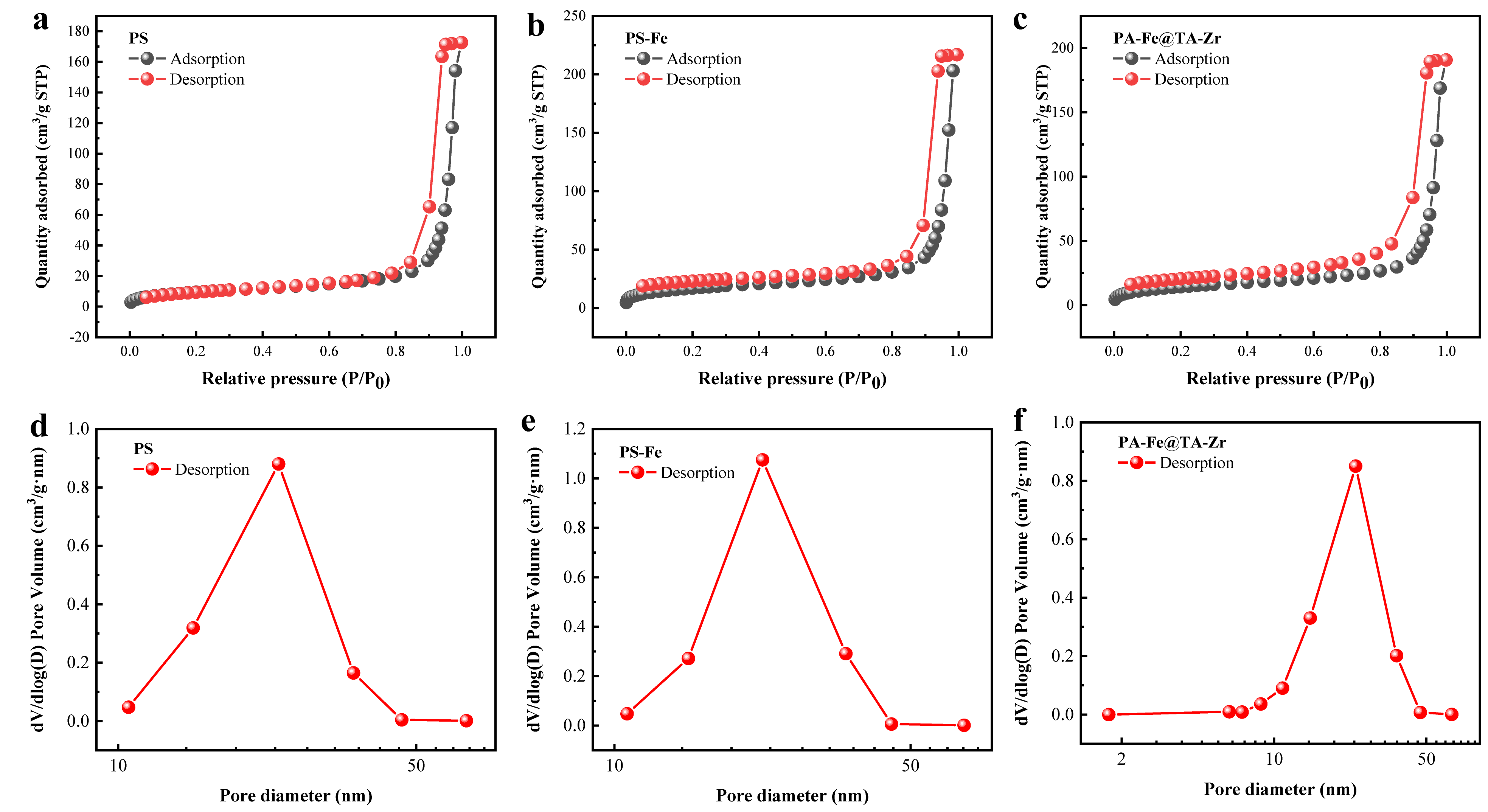


**Figure S2.** (a-c) N_2_ adsorption and desorption isotherms of PS, PS-Fe and PS-Fe@TA-Zr. (d-f) Pore size distribution curves of PS, PS-Fe and PS-Fe@TA-Zr.


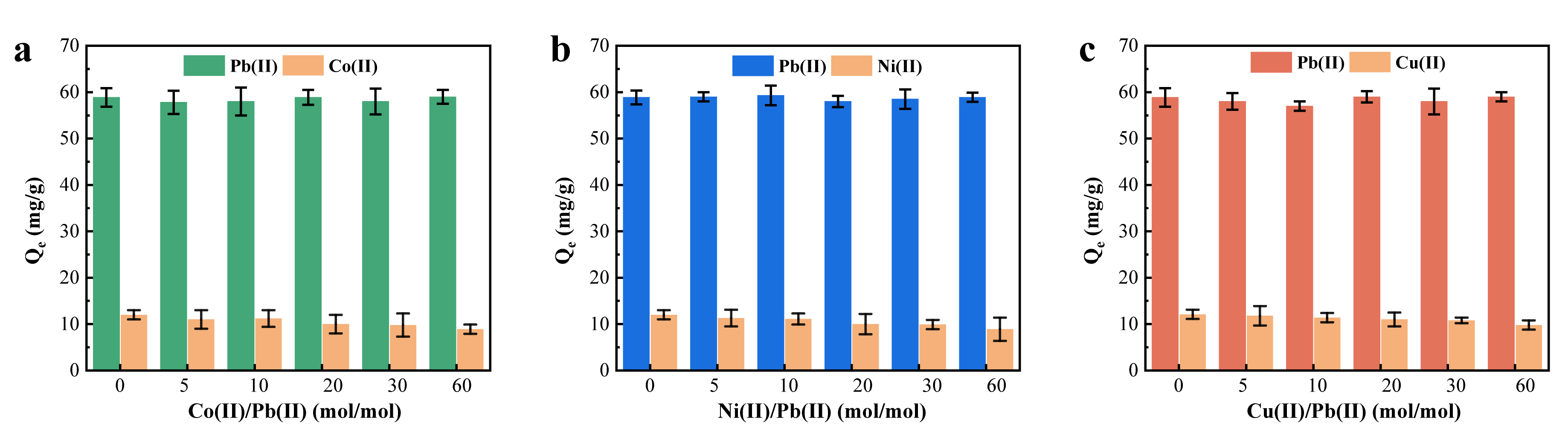


**Figure S3.** (a-c) Effect of competitive comparisons. (a) Co(II); (b) Ni(II); (c) Cu(II). Conditions: 0.1 g/L adsorbents, initial Pb(II) = 10 mg/L, 50 mL solution, pH = 5.5-6.0, 298 K.


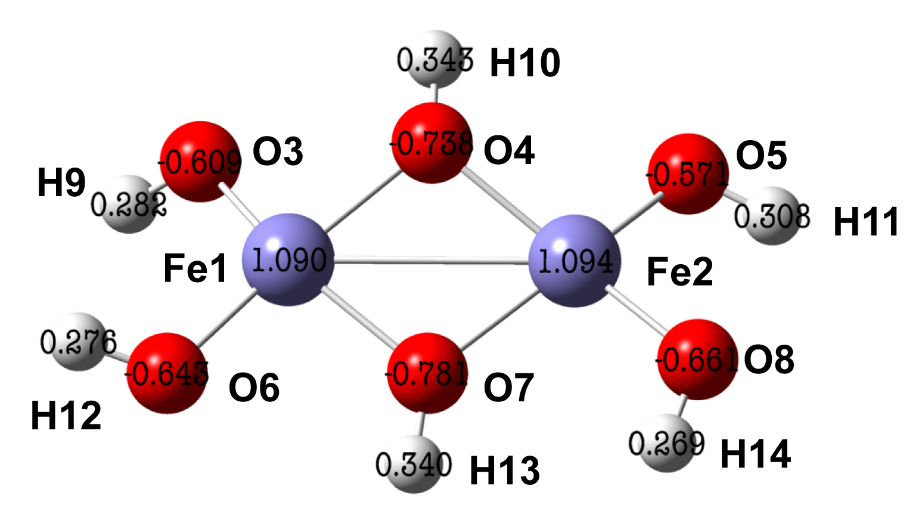


**Figure S4.** The optimal structure and electronic distribution of PS-Fe calculated by DFT.


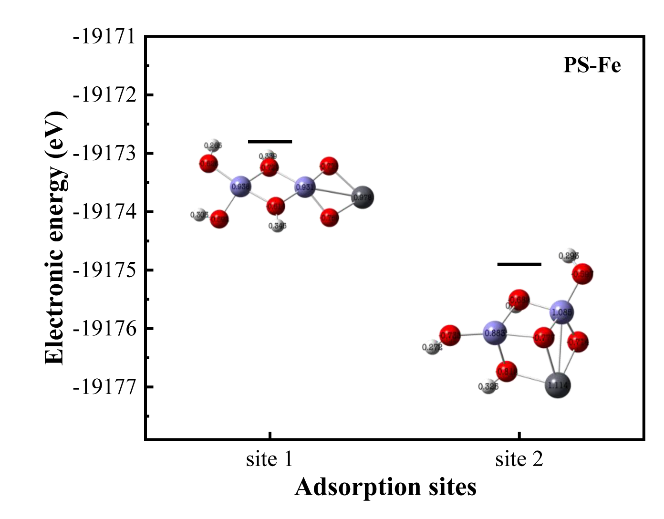


**Figure S5.** The comparison of electronic energy of different adsorption site on PS-Fe.

**
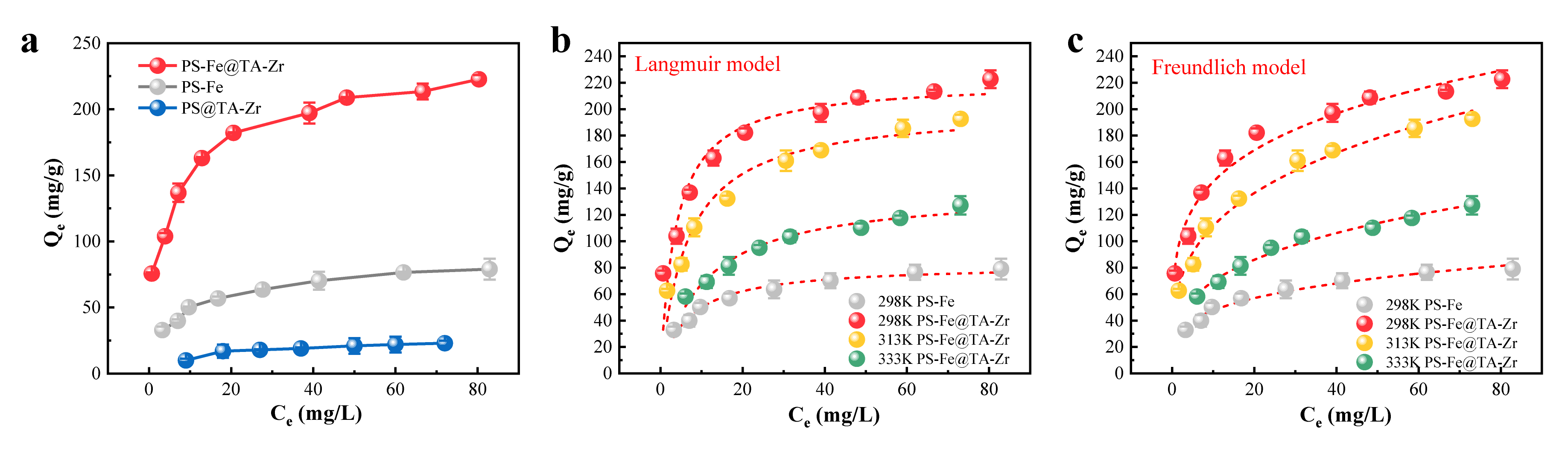
**

**Figure S6.** (a) Adsorption capacity comparison of PS-Fe, PS@TA-Zr and PS-Fe@TA-Zr. (b) sorption isotherm by Langmuir fittings and Freundlich fittings. Conditions: dose 0.1 g/L, 50 mL solution, shake for 12 h, pH = 5.6.


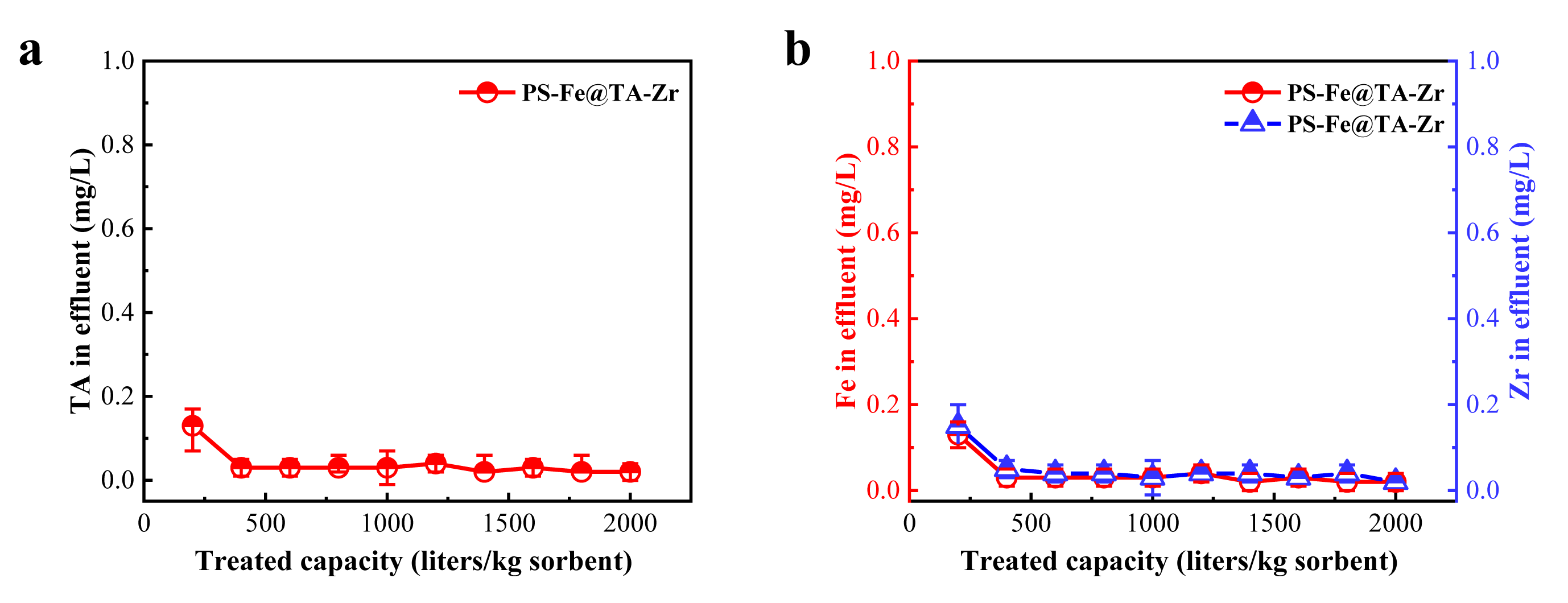


**Figure S7.** (a) TA release and (b) Fe and Zr release of PS-Fe@TA-Zr during the process of column experiment.
